# Disconnect between signalling potency and *in vivo* efficacy of pharmacokinetically optimised biased glucagon-like peptide-1 receptor agonists

**DOI:** 10.1016/j.molmet.2020.100991

**Published:** 2020-04-08

**Authors:** Maria Lucey, Philip Pickford, Stavroula Bitsi, James Minnion, Jan Ungewiss, Katja Schoeneberg, Guy A. Rutter, Stephen R. Bloom, Alejandra Tomas, Ben Jones

**Affiliations:** 1 Section of Investigative Medicine, Imperial College London, London W12 0NN, United Kingdom; 2 Section of Cell Biology and Functional Genomics, Imperial College London, London W12 0NN, United Kingdom; 3 3B Pharmaceuticals GmbH, Berlin, Germany

**Keywords:** Type 2 diabetes, Biased signalling, Glucagon-like peptide-1 receptor, Exendin-4, Trafficking, AUC, area under curve, BSA, bovine serum albumin, cAMP, cyclic adenosine monophosphate, DIO, diet-induced obesity, FITC, fluorescein isothiocyanate, GLP-1RA, glucagon-like peptide-1 receptor agonist, HBSS, Hank's buffered salt solution, HTRF, homogenous time-resolved fluorescence, LC/MSMS, liquid chromatography/tandem mass spectrometry, n.c., not calculable, PBS, phosphate-buffered saline, SEM, standard error of the mean, T2D, type 2 diabetes, TR-FRET, time-resolved Förster resonance energy transfer

## Abstract

**Objective:**

The objective of this study was to determine how pharmacokinetically advantageous acylation impacts on glucagon-like peptide-1 receptor (GLP-1R) signal bias, trafficking, anti-hyperglycaemic efficacy, and appetite suppression.

**Methods:**

In vitro signalling responses were measured using biochemical and biosensor assays. GLP-1R trafficking was determined by confocal microscopy and diffusion-enhanced resonance energy transfer. Pharmacokinetics, glucoregulatory effects, and appetite suppression were measured in acute, sub-chronic, and chronic settings in mice.

**Results:**

A C-terminally acylated ligand, [F^1^,G^40^,K^41^.C16 diacid]exendin-4, was identified that showed undetectable β-arrestin recruitment and GLP-1R internalisation. Depending on the cellular system used, this molecule was up to 1000-fold less potent than the comparator [D^3^,G^40^,K^41^.C16 diacid]exendin-4 for cyclic AMP signalling, yet was considerably more effective *in vivo*, particularly for glucose regulation.

**Conclusions:**

C-terminal acylation of biased GLP-1R agonists increases their degree of signal bias in favour of cAMP production and improves their therapeutic potential.

## Highlights

• Programming of GLP-1R agonists for selective signalling.
• Signal bias allows “low efficacy” agonists to be highly effective *in vivo*.
• GLP-1R endocytosis does not affect pharmacokinetics.

## 1 Introduction

Glucagon-like peptide-1 receptor agonists (GLP-1RAs) are effective agents for the treatment of type 2 diabetes (T2D) and obesity [1]. Their therapeutic effects derive mainly from potentiation of glucose-stimulated insulin secretion and suppression of appetite leading to weight loss, respectively mediated by GLP-1Rs expressed in pancreatic beta cells and anorectic neurons. GLP-1RAs are known to improve renal [2] and cardiovascular outcomes [3] in T2D and reduce mortality [4].

The predominant intracellular signalling intermediate that couples GLP-1R activation to its downstream effects is cyclic adenosine monophosphate (cAMP) [5,6]. However, an updated view of GLP-1R pharmacology highlights the roles of membrane trafficking [7,8] and additional effector proteins such as the β-arrestins [9,10] in the control of amplitude, duration, and subcellular localisation of signalling events to regulate insulin secretion, particularly in the pharmacological setting. Although all clinically approved GLP-1RAs show broadly similar signalling and trafficking characteristics to the endogenous ligand GLP-1(7–36)NH_2_, these can be dramatically altered via sequence modifications close to the ligand N-terminus, as recently demonstrated using analogues of the GLP-1 homologue peptide exendin-4 [11,12]. Specifically, “biased” GLP-1RAs that retain full efficacy for cAMP production but reduced β-arrestin recruitment and endocytic uptake are able to avoid GLP-1R desensitisation and downregulation that ordinarily attenuate glucoregulatory responses *in vivo*.

Studies of orthosteric biased GLP-1R agonism have to date mainly used peptide ligands [11,12], sometimes featuring non-native amino acid substituents [[13], [14], [15]]. While these ligands typically have been engineered for high proteolytic stability, rapid renal elimination [16] means that their half-lives are measured in hours, which is incompatible with a drive to reduce the frequency of injections for patient convenience, comfort, and adherence. The leading GLP-1RAs in current clinical usage have been chemically optimised to allow once-weekly dosing in humans through avoidance of renal clearance [1], for example through conjugation of a fatty acid chain to the peptide that promotes reversible binding to albumin and other plasma proteins that are too large to undergo glomerular filtration. However, these approved compounds show broadly comparable signalling characteristics, and biased GLP-1R agonism has not yet been studied using pharmacokinetically optimised agents.

In this study, we investigated the impact of acylating two oppositely biased GLP-1R agonists, exendin-phe1 (referred to in this manuscript as F^1^-exendin-4) and exendin-asp3 (referred to as D^3^-exendin-4), both with identical amino acid sequences to exendin-4 except for at the first or third N-terminal amino acids, respectively [12]. We found that the introduction of a C-terminal fatty diacid chain to F^1^-exendin-4 exaggerated the degree of bias at the expense of a reduced overall signalling potency. However, when tested *in vivo*, despite an up to ∼1000-fold reduction in signalling potency, the acylated form of F^1^-exendin-4 outperformed that of D^3^-exendin-4 for control of blood glucose over 72 h after a single dose, as well as providing greater glucose control and weight loss with repeated administration.

## 2 Materials and methods

### 2.1 Peptides

Peptides were produced by Wuxi AppTec Co., Ltd., using standard solid phase peptide synthesis. Mass spectrometric confirmation of peptide identity and high-performance liquid chromatographic purity assessment were provided by the manufacturer (all were of >90% purity).

### 2.2 Cell culture

HEK293 cells stably expressing human SNAP-GLP-1R (HEK293-SNAP-GLP-1R cells), HEK293T cells, PathHunter CHO–K1-βarr2-EA-GLP-1R cells (DiscoverX), INS-1 832/3 cells (a gift from Prof. Christopher Newgard, Duke University), and MIN6B1 cells (a gift from Prof. Philippe Halban, University of Geneva) were used in this study and maintained as previously described [12,17]. Full details are provided in the Supplementary Methods.

### 2.3 GLP-1R binding affinity measurement

Equilibrium binding assays were conducted using HEK293-SNAP-GLP-1R labelled with SNAP-Lumi4-Tb (Cisbio, 40 nM). Metabolic inhibitors (20 mmol/L 2-deoxygucose and 10 mmol/L NaN_3_) [18] were used to prevent GLP-1R internalisation during agonist binding. Cells were treated with exendin [[9], [10], [11], [12], [13], [14], [15], [16], [17], [18], [19], [20], [21], [22], [23], [24], [25], [26], [27], [28], [29], [30], [31], [32], [33], [34], [35], [36], [37], [38], [39]]-FITC in competition with a range of concentrations of unlabelled peptide for 24 h at 4 °C before measurement of binding by TR-FRET [17]. The baseline-subtracted TR-FRET ratio was used to quantify binding and equilibrium binding constants (K_d_) using Prism 8 (GraphPad Software). Full details are provided in the Supplementary Methods.

### 2.4 Cyclic AMP responses

Cells were stimulated with agonist for the indicated time period in their respective growth media. FBS was used when indicated. Assays were conducted at 37 °C without phosphodiesterase inhibitors except for INS-1 832/3 and MIN6B1 cells, where 3-isobutyl-1-methylxanthine (IBMX) was added at 500 μM to detect ligand-induced responses in these less highly coupled cell models. At the end of the incubation period, the cells were lysed and cAMP was determined by homogenous time-resolved fluorescence (HTRF, cAMP Dynamic 2 kit, Cisbio).

### 2.5 PathHunter β-arrestin recruitment assay

Cells were stimulated for 30 min in growth medium without FBS at 37 °C. The assay was terminated by the addition of PathHunter detection reagents and the luminescent signal was read from each well.

### 2.6 NanoBiT complementation assays

The plasmids for mini-G_s_, -G_i_, and -G_q_, each carrying an N-terminal LgBiT tag [19], were a gift from Prof. Nevin Lambert, Medical College of Georgia. The plasmid for β-arrestin-2 fused at the N-terminus to LgBiT was obtained from Promega Custom Assay Services (plasmid CS1603B118). The SmBiT was cloned in frame at the C-terminus of the GLP-1R by substitution of the Tango sequence on a FLAG-tagged GLP-1R-Tango expression vector [20], a gift from Dr. Bryan Roth, University of North Carolina (Addgene plasmid # 66295). HEK293T cells in 12-well plates were co-transfected for 24 h using Lipofectamine 2000 and the following quantities of DNA: 0.5 μg each of GLP-1R-SmBit and LgBit-mini-G, or 0.05 μg each of GLP-1R-SmBit and LgBit-mini-G with 0.9 μg empty vector DNA (pcDNA3.1). Cells were resuspended in Nano-Glo dilution buffer + fumarazine (Promega) diluted 1:20 and seeded in 96-well half area white plates. Baseline luminescence was measured over 5 min using a Flexstation 3 microplate reader at 37 °C before the addition of ligand or vehicle. Responses were normalised to the average baseline.

### 2.7 PKA activation FRET assay

This assay was conducted as previously described [17]. Briefly, HEK293-SNAP-GLP-1R cells were transfected with PKA activation FRET biosensor AKAR4-NES (a gift from Dr. Jin Zhang, Addgene plasmid #61620) for 36 h, and FRET signal was recorded before and after ligand addition and expressed ratiometrically after normalisation to well baseline. Full details are provided in the Supplementary Methods.

### 2.8 Confocal microscopy

HEK293-SNAP-GLP-1R cells seeded on coverslips were labelled with SNAP-Surface 549 (New England Biolabs) prior to stimulation with the indicated agonist (100 nM) for 30 min, after which the cells were washed in PBS, fixed with 4% paraformaldehyde, mounted in Diamond Prolong mounting medium with DAPI, imaged with a Zeiss LSM780 inverted confocal microscope with a 63x/1.4 numerical aperture oil-immersion objective, and analysed in Fiji.

### 2.9 Animal studies

All of the animal procedures were approved by the British Home Office under the UK Animal (Scientific Procedures) Act 1986 (Project License PB7CFFE7A). Animal husbandry details are provided in the Supplementary Methods.

### 2.10 Pharmacokinetic studies

Mice were injected intraperitoneally (IP) with 100 nmol/kg agonist and blood samples were obtained by venesection into lithium heparin-coated capillary tubes. Plasma exendin concentrations were measured using a fluorescent enzyme immunoassay (Phoenix Pharmaceuticals) that recognises the C-terminus of exendin-4 and has previously been used to measure the pharmacokinetics of N-terminally modified exendin-4 analogues [11,12]. As the manufacturer also reports full cross-reactivity with lixisenatide, a peptide with a similar amino acid sequence to exendin-4 except for a C-terminal modification featuring a hexalysine extension, we suspected it may also recognise the acylated peptides used in this study. At 0.1 nM, we found 50% cross-reactivity of acylated exendin-4 analogues compared to exendin-4 when spiked into mouse plasma, confirming a fully intact exendin-4 C-terminus is not mandatory for this assay. This correction factor was therefore applied to correct for reduced recovery of acylated peptides.

### 2.11 *ES*calate assay

The *ES*calate assay was conducted as previously described [21]. Full details are provided in the Supplementary Methods.

### 2.12 Glucose tolerance testing

Mice were fasted for 4–5 h before the test. Bodyweight-adjusted doses of glucose (2 g/kg) were IP injected with or without agonist prepared within the same injector as indicated. Blood samples were obtained immediately prior to injection and at 20-min intervals thereafter, and glucose was measured using a handheld glucose meter (GlucoRx).

### 2.13 Food intake studies

For sub-chronic studies, mice were fasted overnight and diet was returned to the cage immediately after IP injection of agonist, with cumulative intake determined by weighing. For the chronic study, mice were fasted for 4–5 h during the light phase and diet was returned to the cage immediately after IP injection of agonist at the beginning of the dark phase.

### 2.14 Alpha and beta cell mass quantification

At the end of the agonist administration period, mice were sacrificed and the pancreata were dissected and fixed in 4% PFA for 24 h. Insulin- and glucagon-positive areas were quantified as previously described [22] and expressed relative to the total pancreas area imaged. Full details are provided in the Supplementary Methods.

### 2.15 Statistical analyses

Quantitative data were analysed using Prism 8.0 (GraphPad Software). In cell culture experiments, technical replicates were averaged so that each individual experiment was treated as one biological replicate. Dose responses were analysed using 4-parameter logistic fits, with constraints imposed as appropriate. Bias analyses were conducted as previously described [12,23]. Statistical comparisons were made by t-test or ANOVA as appropriate, with paired or matched designs used depending on the experimental design. Mean ± standard error of mean (SEM) or individual replicates are displayed throughout. Statistical significance was inferred if p < 0.05.

## 3 Results

### 3.1 Impact of C-terminal acylation on *in vitro* pharmacology and trafficking of biased GLP-1RAs

**Figure 1:**
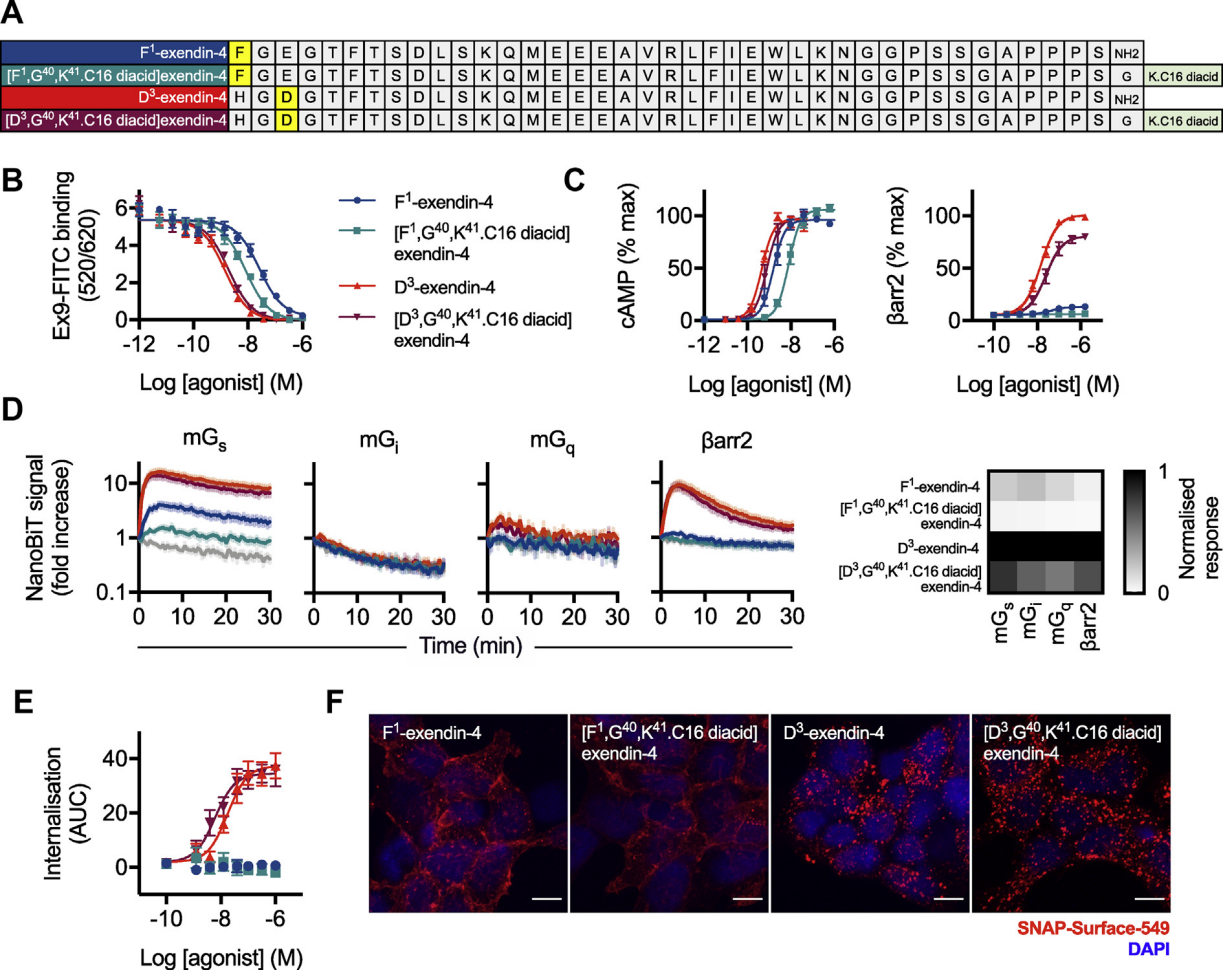
***In vitro* pharmacological properties of acylated biased GLP-1RAs.** (**A**) Amino acid sequences in single letter code for the peptides used in this study, indicating the position of C16 diacid conjugation. (**B**) Equilibrium binding of each ligand in competition with exendin(9–39)-FITC in HEK293-SNAP-GLP-1R cells, *n* = 5. (**C**) Cyclic AMP (cAMP) production and β-arrestin-2 (βarr2) recruitment in CHO–K1-βarr2-EA-GLP-1R cells, 30-min incubation, *n* = 6, 4-parameter logistic fit of pooled data shown. (**D**) NanoBiT recruitment assays conducted in HEK293T cells transiently transfected with GLP-1R-SmBiT and miniG_s_-LgBiT (mG_s_, *n* = 10), miniG_i_-LgBiT (mG_i_, *n* = 4), miniG_q_-LgBiT (mG_q_, *n* = 4), or LgBiT-β-arrestin-2 (βarr2, *n* = 6). Vehicle response in light grey. Note the logarithmic scale for the y-axis. The heatmap shows AUC quantifications for each pathway normalised to the most efficacious ligand. (**E**) GLP-1R endocytosis measured in HEK293-SNAP-GLP-1R cells by DERET, indicated as AUC from kinetic traces shown in Supplementary Figure 1F, *n* = 5, 4-parameter logistic fit of pooled data shown. (**F**) Representative maximum intensity projection images, showing GLP-1R endocytosis in HEK293-SNAP-GLP-1R cells labelled with SNAP-Surface 549 prior to stimulation with indicated agonist (100 nM) for 30 min, *n* = 3, scale bar: 8 μm ∗p < 0.05 by statistical test indicated in the text. Error bars indicate SEM.

The sequences of F^1^-exendin-4, D^3^-exendin-4, and their acylated equivalents [F^1^,G^40^,K^41^⁰.C16 diacid]exendin-4 and [D^3^,G^40^,K^41^.C16 diacid]exendin-4 are shown in Figure 1A and Supplementary Figure 1A. The C16 hexadecanedioic acid moiety was inserted at the peptide C-terminus after a GK linker. This position was chosen to avoid the potential for fatty acids to interfere with putative interactions made by the peptide N-terminus with the GLP-1R, which are important for inducing signal bias. The equilibrium affinity of [D^3^,G^40^,K^41^.C16 diacid]exendin-4 was similar to that of D^3^-exendin-4 (Figure 1B, log K_d_ −8.9 ± 0.1 vs −9.1 ± 0.1, respectively, p > 0.05 by one-way randomised block ANOVA with Sidak's test), whereas [F^1^,G^40^,K^41^.C16 diacid]exendin-4 showed higher affinity for the receptor than its non-acylated counterpart (log K_d_ −8.3 ± 0.0 vs −7.8 ± 0.0, respectively, p < 0.05 by one-way randomised block ANOVA with Sidak's test).

Non-acylated F^1^-exendin-4 is known to display markedly reduced recruitment of β-arrestin-1 and -2, whereas non-acylated D^3^-exendin-4 is a full agonist in these pathways, with both ligands being full agonists for cAMP [12]. To determine how acylation affects this established pattern of signal bias, we measured cAMP and β-arrestin-2 responses in PathHunter CHO–K1-βarr2-EA-GLP-1R cells. The expected signalling pattern was preserved (Figure 1C, Table 1), although both C16 ligands showed reduced efficacy for β-arrestin-2 recruitment compared to their non-acylated equivalents. Despite increased binding affinity, cAMP potency for [F^1^,G^40^,K^41^.C16 diacid]exendin-4 was reduced compared to that of F^1^-exendin-4. Due to the undetectable β-arrestin-2 response with [F^1^,G^40^,K^41^.C16 diacid]exendin-4, quantification of signal bias was not possible either using the most commonly used approach based on a modified form of the operational model of agonism [24,25], or an alternative method designed to aid bias quantification with extremely low efficacy agonists [23] (Supplementary Figure 1B).

**Table 1:** **Binding, signalling, and internalisation parameter estimates for the ligands in this study.** Signalling parameter measures were determined as follows: an initial 4-parameter fit was constructed for all full agonists, with globally constrained basal response E_max_ and Hill slope, to establish the maximal response for the assay (note that for β-arrestin-2 measurement, D^3^-exendin-4 was the only full agonist). Individual responses were normalised to the assay maximum and parameter estimates for each assay recalculated by 4-parameter fitting with globally constrained basal response and Hill slope, but no constraint to E_max_. For internalisation, curve fitting was performed similarly but without prior normalisation to a maximal response. Note that for β-arrestin-2, meaningful estimates of [F^1^,G^40^,K^41^.C16 diacid]exendin-4 could not be calculated (n.c.), as was also the case for both F^1^-exendin-4 and [F^1^,G^40^,K^41^.C16 diacid]exendin-4 for internalisation. Average ± SEM values are reported.

	CHO–K1-βarr2-EA-GLP-1R				INS-1 832/3		MIN6B1		HEK293-SNAP-GLP-1R				
	cAMP		βarr2		cAMP		cAMP		cAMP (1% FBS)		Internalisation		Binding
	pEC_50_ (M)	E_max_ (% max)	pEC_50_ (M)	E_max_ (% max)	pEC_50_ (M)	E_max_ (% max)	pEC_50_ (M)	E_max_ (% max)	pEC_50_ (M)	E_max_ (% max)	pEC_50_ (M)	E_max_ (AUC)	pK_d_ (M)
D^3^-exendin-4	9.3 ± 0.0	96 ± 4	7.8 ± 0.1	100	9.0 ± 0.3	100 ± 2	11.0 ± 0.3	101 ± 2	10.6 ± 0.3	96 ± 2	7.8 ± 0.1	38 ± 4	9.1 ± 0.1
[D^3^,K^40^.C16 diacid]exendin-4	9.1 ± 0.1	100 ± 4	7.6 ± 0.2	81 ± 2	8.7 ± 0.2	101 ± 2	10.8 ± 0.2	97 ± 1	10.4 ± 0.2	97 ± 3	8.3 ± 0.2	35 ± 4	8.9 ± 0.1
F^1^-exendin-4	8.8 ± 0.1	97 ± 2	7.3 ± 0.1	14 ± 1	8.4 ± 0.3	97 ± 3	9.2 ± 0.4	97 ± 5	9.0 ± 0.1	83 ± 1	n.c.	n.c	7.8 ± 0.0
[F^1^,K^40^.C16 diacid]exendin-4	8.0 ± 0.1	107 ± 3	n.c.	n.c	7.5 ± 0.2	41 ± 4	7.9 ± 0.3	98 ± 7	8.2 ± 0.1	49 ± 3	n.c.	n.c	8.3 ± 0.0

Signalling potencies for cAMP at rat and mouse GLP-1Rs was also tested in INS-1 832/3 and MIN6B1 insulinoma cells, respectively (Supplementary Figure 1C and C, Table 1). In these cells, which express the GLP-1R endogenously, the potency shift with [F^1^,G^40^,K^41^.C16 diacid]exendin-4 was more pronounced (up to 1000-fold reduction in MIN6B1 compared to [D^3^,G^40^,K^41^.C16 diacid]exendin-4), accompanied by reduced efficacy in INS-1 832/3 cells. These differences are more marked than for cells with GLP-1R exogenously expressed at high levels, for example, the experiments shown in Figure 1C.

We also used NanoBiT complementation to monitor dynamic interactions of GLP-1R with β-arrestin-2 [26] and mini-G_s_, -G_i_, and -G_q_ protein probes [19] after stimulation with a maximal concentration of each ligand (Figure 1D; individual AUC quantifications are shown in Supplementary Figure 1D). Mini-G_q_ and -G_i_ recruitment responses were detectable in some cases but of low magnitude, confirming that G_s_ is the preferred G protein coupled to GLP-1R activation. Both F^1^ ligands showed markedly reduced efficacy for G_s_ recruitment compared to D^3^ ligands, and acylation was associated with a modest reduction in recruitment efficacy for each effector (see the heatmap in Figure 1D). Comparison of maximal responses in HEK293 cells for mini-G_s_ recruitment, cAMP, and protein kinase A (PKA) activation highlighted how amplification within this pathway translates low efficacy G protein recruitment to high efficacy PKA signalling with F^1^ ligands in the context of virtually absent β-arrestin-2 recruitment (Supplementary Figure 1E).

Next, the four ligands were compared in HEK293-SNAP-GLP-1R cells for their propensity to induce GLP-1R internalisation as measured by diffusion-enhanced resonance energy transfer (DERET) [27]. D^3^-exendin-4 and [D^3^,G^40^,K^41^.C16 diacid]exendin-4 induced rapid internalisation, whereas none was detectable with either type of F^1^ ligand (Figure 1E, Supplementary Figure 1F, Table 1). These results were corroborated by confocal microscopy, which showed that surface-labelled SNAP-GLP-1R was predominantly relocated to punctate endosomal organelles after D^3^-exendin-4 and [D^3^,G^40^,K^41^.C16 diacid]exendin-4 treatment, but remained mainly at the plasma membrane with F^1^-exendin-4 and [F^1^,G^40^,K^41^.C16 diacid]exendin-4 (Figure 1F).

This initial *in vitro* characterisation demonstrated that the C16 diacid C-terminal conjugation was well tolerated by D^3^-exendin-4. Some differences were however observed with the pharmacology of [F^1^,G^40^,K^41^.C16 diacid]exendin-4, which showed increased binding affinity but decreased signalling efficacy compared to F^1^-exendin-4, thereby magnifying signalling differences between the two oppositely biased ligands.

### 3.2 Acylated biased GLP-1RAs show prolonged pharmacokinetics

Acylation prolongs peptide pharmacokinetics by promoting reversible binding to plasma proteins that are too large to undergo glomerular filtration, such as albumin. To determine the extent of [F^1^,G^40^,K^41^.C16 diacid]exendin-4, [D^3^,G^40^,K^41^.C16 diacid]exendin-4, and the similarly designed [G^40^,K^41^.C16 diacid]exendin-4 (Supplementary Figure 2A) binding to plasma proteins, we used the *ES*calate equilibrium shift assay [21]. The results indicated that each acylated ligand exhibited a high and similar degree of binding to mouse and human plasma proteins (Table 2, Supplementary Tables 1 and 2). In keeping with this, the presence of serum during the incubation did not differentially affect the signalling potency for each C16 ligand in GLP-1R cAMP assays compared to its non-acylated comparator (Supplementary Figure 2B); potency was in fact higher when serum was present for both acylated and non-acylated ligands, presumably due to reduced adsorption of peptide onto the microplate plastic surface. In this system, an approximately 100-fold potency difference between [F^1^,G^40^,K^41^.C16 diacid]exendin-4 and [D^3^,G^40^,K^41^.C16 diacid]exendin-4 was observed, along with reduced efficacy for the former (Table 1). The pharmacology of [G^40^,K^41^.C16 diacid]exendin-4 was evaluated in CHO–K1-βarr2-EA-GLP-1R cells, revealing preserved cAMP signalling potency with a clear reduction in β-arrestin-2 recruitment efficacy compared to exendin-4, conforming to the patterns observed with the F^1^ and D^3^ ligand pairs (Supplementary Figure 2C).

**Table 2:** ***ES*calate results demonstrating binding of each ligand to human and mouse plasma proteins**. Data are expressed as mean ± SEM of the percentage of peptide unbound to plasma proteins determined in duplicate.

Compound	Unbound fraction (f_u_)	
	Human plasma	Mouse plasma
[G^40^,K^41^.C16 diacid]exendin-4	2.3% ± 1.0%	2.6% ± 0.1%
[F^1^,G^40^,K^41^.C16 diacid]exendin-4	2.1% ± 0.1%	2.4% ± 0.3%
[D^3^,G^40^,K^41^.C16 diacid]exendin-4	2.2% ± 0.1%	2.1% ± 0.3%

**Figure 2:**
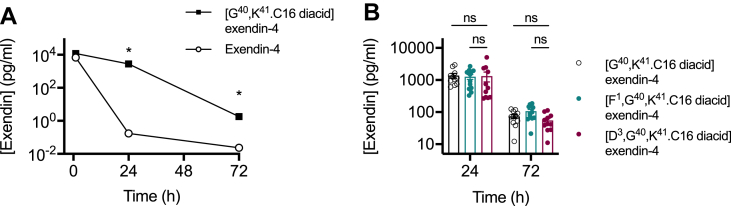
**Pharmacokinetic properties of acylated biased GLP-1RAs.** (**A**) Plasma concentration of exendin-4 or [G^40^,K^41^.C16 diacid]exendin-4 (both referred to as Exendin on the y-axis) in male C57Bl/6 mice after a single intraperitoneal injection of 100 nmol/kg agonist, *n* = 8 per treatment, two-way repeat measures ANOVA of log-transformed data with Sidak's test. (**B**) Plasma concentration of [G^40^,K^41^.C16 diacid]exendin-4 (*n* = 10), [F^1^,G^40^,K^41^.C16 diacid]exendin-4 (*n* = 12), and [D^3^,G^40^,K^41^.C16 diacid]exendin-4 (*n* = 11) in male C57Bl/6 mice after a single intraperitoneal injection of 100 nmol/kg agonist, two-way repeat measures ANOVA on log-transformed data with Sidak's test. In this experiment, mice (*n* = 8) injected with vehicle recorded a nominal exendin plasma concentration of 5.8 pg/ml at 72 h, reflecting non-specific signalling at the lower limit of the assay. ∗p < 0.05 by statistical test indicated in the text. Error bars indicate SEM.

In keeping with the anticipated pharmacokinetic effect resulting from binding to plasma proteins, after a single injection in mice, [G^40^,K^41^.C16 diacid]exendin-4 remained detectable in the circulation for at least 72 h, in contrast to non-acylated exendin-4 (Figure 2A). We considered the possibility of whether GLP-1R-mediated clearance may play a role in pharmacokinetics once glomerular filtration is no longer a primary route of elimination in a form of target-mediated drug disposal (TMDD) [28,29], but the absence of large differences in circulating concentrations of all three acylated ligands 24 and 72 h post-dosing argues against this possibility (Figure 2B).

Therefore, conjugation of a C16 diacid to the C-terminus of exendin-F^1^ and exendin-D^3^ results in a pair of pharmacokinetically advantageous and oppositely biased GLP-1RAs, allowing convenient assessment of the impact of signal bias and trafficking over multiple days.

### 3.3 Metabolic effects of biased GLP-1RAs are preserved after acylation

The primary therapeutic actions of GLP-1RAs are to improve glucose regulation and reduce appetite. We trialled a series of doses of [F^1^,G^40^,K^41^.C16 diacid]exendin-4 and [D^3^,G^40^,K^41^.C16 diacid]exendin-4 in lean C57Bl/6 mice to assess their *in vivo* performance over 72 h. Both ligands exhibited effective glucoregulatory properties (Supplementary Figure 3A), although [F^1^,G^40^,K^41^.C16 diacid]exendin-4 was less effective at 10 nmol/kg, which could reflect its reduced signalling potency. However, when reassessed at 72 h, [D^3^,G^40^,K^41^.C16 diacid]exendin-4 no longer exhibited any detectable anti-hyperglycaemic efficacy, whereas both of the higher [F^1^,G^40^,K^41^.C16 diacid]exendin-4 doses remained effective. The acute anorectic effect of [D^3^,G^40^,K^41^.C16 diacid]exendin-4 was somewhat greater than that of [F^1^,G^40^,K^41^.C16 diacid]exendin-4, particularly at the 10 nmol/kg dose (Supplementary Figure 3B), although, interestingly, by 72 h, the net calorie intake deficit with [F^1^,G^40^,K^41^.C16 diacid]exendin-4 at the highest dose was greater than that of [D^3^,G^40^,K^41^.C16 diacid]exendin-4. A dose response analysis of these data is displayed in Supplementary Figure 3C.

**Figure 3:**
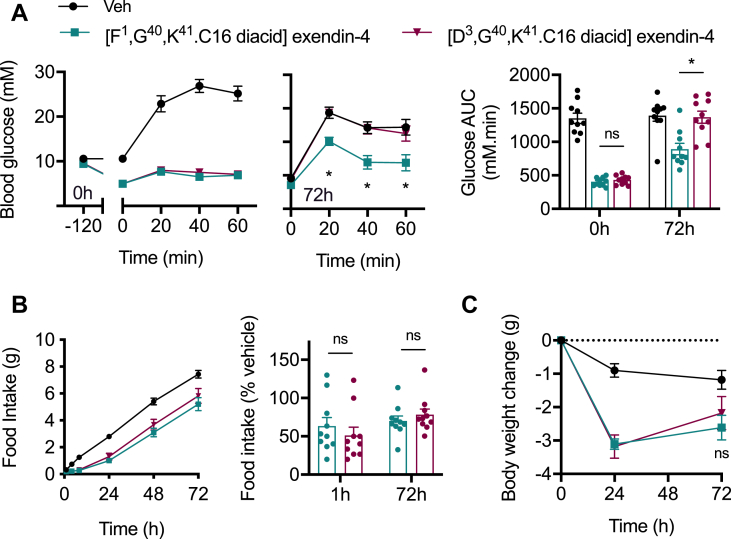
**Sub-chronic effects of acylated biased GLP-1RAs in diet-induced obese mice.** (**A**) Intra-peritoneal glucose tolerance tests (IPGTT, 2 g/kg glucose) conducted 2 or 72 h after IP administration of indicated -C16 agonist (10 nmol/kg) or vehicle (saline) in male diet-induced obese (DIO) C57Bl/6 mice, *n* = 10/group, time points and AUCs both compared by two-way repeat measures ANOVA with Tukey's test, with comparisons between [F^1^,G^40^,K^41^.C16 diacid]exendin-4 and [D^3^,G^40^,K^41^.C16 diacid]exendin-4 are shown. (**B**) Cumulative food intake in male DIO C57Bl/6 mice after IP administration of indicated -C16 agonist (10 nmol/kg) or vehicle (saline), *n* = 10/group, effect of each agonist to reduce food intake relative to vehicle at 1 and 72 h is shown separately. (**C**) Body weight change in the study shown in (B), two-way repeat measures ANOVA with Tukey's test, comparison between [F^1^,G^40^,K^41^.C16 diacid]exendin-4 and [D^3^,G^40^,K^41^.C16 diacid]exendin-4 at 72 h is shown on the graph. ∗p < 0.05 by statistical test indicated in the text. Error bars indicate SEM.

We also tested both compounds at an intermediate dose in obese mice fed a high-fat diet for 3 months. Consistent patterns were observed, with equal glucose lowering seen 2 h after dosing but a glycaemic advantage for [F^1^,G^40^,K^41^.C16 diacid]exendin-4 clearly demonstrated at 72 h (Figure 3A). The anorectic differences were more subtle than in lean mice, with non-significant trends observed 1 and 72 h after dosing (Figure 3B). A non-significant trend was also seen for body weight loss, with [F^1^,G^40^,K^41^.C16 diacid]exendin-4-treated mice losing slightly more weight at 72 h than those treated with [D^3^,G^40^,K^41^.C16 diacid]exendin-4 (Figure 3C).

The apparent greater efficacy with [F^1^,G^40^,K^41^.C16 diacid]exendin-4 at 72 h suggests that the impact of its biased pharmacology is preserved *in vivo* despite its considerably lower net signalling potency. As previously observed, these effects primarily concerned its glucoregulatory properties.

### 3.4 Sustained administration of acylated biased GLP-1RAs

**Figure 4:**
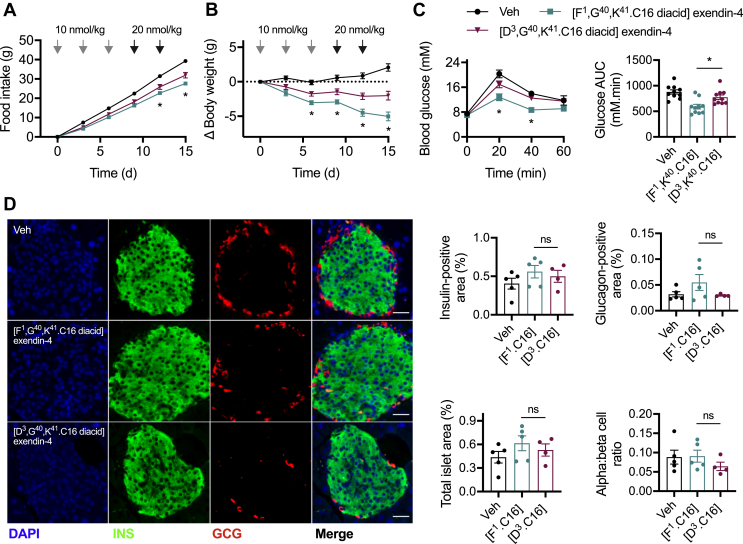
**Effects of chronic administration of biased GLP-1RAs in diet-induced obese mice.** (**A**) Cumulative food intake in male DIO C57Bl/6 mice after IP administration of -C16 agonist every 72 h or vehicle (saline), *n* = 10/group, two-way repeat measures ANOVA with Tukey's test showing comparison between [F^1^,G^40^,K^41^.C16 diacid]exendin-4 and [D^3^,G^40^,K^41^.C16 diacid]exendin-4. The dose was doubled after the first 3 injections to counteract adaptive mechanisms typically seen in rodents treated with GLP-1RAs that limit weight loss [[30], [31], [32]]. (**B**) Change in body weight during the study shown in (A) with two-way repeat measures ANOVA with Tukey's test showing comparison between [F^1^,G^40^,K^41^.C16 diacid]exendin-4 and [D^3^,G^40^,K^41^.C16 diacid]exendin-4. (**C**) IPGTT (2 g/kg glucose) conducted 72 h after the final agonist dose, time points compared by two-way repeat measures ANOVA with Tukey's test (comparison between [F^1^,G^40^,K^41^.C16 diacid]exendin-4 and [D^3^,G^40^,K^41^.C16 diacid]exendin-4 is shown) and AUC compared by one-way ANOVA with Tukey's test (comparison between [F^1^,G^40^,K^41^.C16 diacid]exendin-4 and [D^3^,G^40^,K^41^.C16 diacid]exendin-4 is shown). (**D**) Representative immunohistochemical images showing islet insulin (INS) and glucagon (GCG) staining after 15 d agonist administration, with quantification of insulin-positive area, glucagon-positive area, and alpha:beta cell area ratio from *n* = 5 (vehicle, [F^1^,G^40^,K^41^.C16 diacid]exendin-4) or *n* = 4 ([D^3^,G^40^,K^41^.C16 diacid]exendin-4) mice; comparison by one-way ANOVA with Tukey's test (comparisons between [F^1^,G^40^,K^41^.C16 diacid]exendin-4 and [D^3^,G^40^,K^41^.C16 diacid]exendin-4 are shown). Note that technical failure meant that the pancreas from the fifth [D^3^,G^40^,K^41^.C16 diacid]exendin-4-treated mouse could not be processed. ∗p < 0.05 by statistical test indicated in the text. Error bars indicate SEM.

We conducted a repeated administration study in high-fat diet-induced obese mice to compare the therapeutic effects of [F^1^,G^40^,K^41^.C16 diacid]exendin-4 and [D^3^,G^40^,K^41^.C16 diacid]exendin-4 in a chronic setting. Mice were injected every 72 h over 15 d; the dose was doubled after the first 3 injections to counteract adaptive mechanisms typically seen in rodents treated with GLP-1RAs, which limit weight loss [[30], [31], [32]]. Over the course of the study, the trends observed in the single dose administration studies became more apparent, with a progressively greater anorectic effect observed with [F^1^,G^40^,K^41^.C16 diacid]exendin-4, along with a corresponding divergence in body weight (Figure 4A,B). Glucose tolerance assessed 72 h after the final dose confirmed the expected advantage of [F^1^,G^40^,K^41^.C16 diacid]exendin-4 over [D^3^,G^40^,K^41^.C16 diacid]exendin-4 (Figure 4C). In a separately conducted study in which the glycaemic effects of each ligand were recapitulated (Supplementary Figure 4A), a non-significant trend toward increased beta and alpha cell mass was observed in the [F^1^,G^40^,K^41^.C16 diacid]exendin-4-treated mice, with no change in the ratio of alpha to beta cells (Figure 4D).

Thus, the metabolic benefits of the biased GLP-1RA [F^1^,G^40^,K^41^.C16 diacid]exendin-4 were preserved on repeated administration and became progressively more apparent over time.

## 4 Discussion

In the present study, we developed and tested two oppositely biased GLP-1RAs bearing C-terminal C16 diacid chains, which confer extended pharmacokinetic profiles. The major observation from our data is that, despite at least a large reduction in cAMP signalling potency (10- to 1000-fold depending on the cell system used), [F^1^,G^40^,K^41^.C16 diacid]exendin-4 was markedly more efficacious *in vivo* than oppositely biased [D^3^,G^40^,K^41^.C16 diacid]exendin-4. This finding builds on earlier reports highlighting the advantageous glucoregulatory properties of lower affinity biased GLP-1RAs that favour cAMP signalling over β-arrestin recruitment [11,12], but with a substantially greater disconnect between acute *in vitro* potency and anti-hyperglycaemic properties than for earlier compounds.

Some interesting *in vitro* observations arise from this work, particularly regarding comparisons of acylated vs non-acylated ligands. First, we found that the binding affinity of [F^1^,G^40^,K^41^.C16 diacid]exendin-4 was approximately 3 times greater than that of non-acylated exendin-F^1^, yet its cAMP signalling potency was between 5 and 20 times (for CHO–K1 and MIN6B1 cells, respectively) lower. One possible explanation for this is disruption of normal ligand–receptor interactions by the acyl chain in a manner that permits ligand binding but orientates the ligand so that its N-terminus fails to optimally engage with the receptor core, leading to reduced levels of activation. Structural and computational studies will be required to reveal if is this is the case, but may be hampered by the lack of confidence about the position of the exendin-4 C-terminus when bound to GLP-1R [33]. An alternative possibility, tentatively supported by recent reports that active GLP-1Rs segregate into cholesterol-rich nanodomains within the plasma membrane [17,34], is that fatty acid preferentially directs the ligand to bind to subpopulations of GLP-1Rs residing in membrane regions with reduced enrichment of signalling effectors on the cytoplasmic side. Should this be the case, the fact that nanodomain clustering is more extensive with exendin-D^3^ than exendin-F^1^ [17] could support our observation of greater potency loss with [F^1^,G^40^,K^41^.C16 diacid]exendin-4 than [D^3^,G^40^,K^41^.C16 diacid]exendin-4.

The addition of a C16 diacid moiety, as expected from other ligands tested as part of the preclinical development of C18 diacid-containing semaglutide [35,36], provided a high degree of binding to both mouse and human plasma proteins. The slightly lower affinity of the investigated compounds (2.08%–2.33% in human plasma) compared to liraglutide containing a C16 fatty acid moiety (0.51% in human plasma [21]) may be attributable to the shorter linker between the peptide and albumin binding group. In combination with resistance of exendin peptides to degradation by a variety of enzymes including dipeptidyl dipeptidase-4 and neutral endopeptidases [37], the two primary elimination routes for GLP-1 peptides are avoided, providing a basis for substantially extended pharmacokinetics in mice, with even longer protraction expected to be obtained in humans. We note a discrepancy between the absolute plasma levels recorded at 72 h for [G^40^,K^41^.C16 diacid]exendin-4 in the separately conducted experiments presented in Figure 2A,B, which we suspect results from assay-to-assay variability.

Our evaluation of the pharmacodynamic performance of biased GLP-1RAs found clear evidence of improved anti-hyperglycaemic efficacy for [F^1^,G^40^,K^41^.C16 diacid]exendin-4. This is despite substantially reduced acute signalling potency of this molecule, highlighting how standard *in vitro* approaches may fail to identify optimal agonist characteristics. Although coupling to G_s_ recruitment was markedly reduced, it can be speculated that the virtual absence of β-arrestin-2 recruitment and GLP-1R internalisation allow prolongation of signalling despite an initial deficit as previously described [12]. Moreover, inherent amplification in the G_s_/cAMP/PKA pathway appears to allow low efficacy G_s_ recruitment to translate to full amplitude responses in downstream pathways. Interestingly, recent work suggested that β-arrestins play a minimal role in controlling GLP-1R endocytosis [17] and may not diminish acute insulin secretory responses from pancreatic beta cells [9,38]. However, the latter observation needs to be evaluated under conditions of sustained exposure to pharmacokinetically optimised GLP-1RAs in line with the appropriate timescales for glucoregulatory benefits of biased GLP-1RAs, which tend to emerge after a number of hours. Interestingly, GLP-1R internalisation was reported to be G_q_-dependent [39], and with this in mind, it may be relevant that we detected G_q_ recruitment to GLP-1R after treatment with fast internalising asp3 ligands. |

It is notable that the differences in physiological response entrained by oppositely biased ligands concerned primarily their glucoregulatory effects, with smaller differences observed in feeding behaviour. A similar pattern was previously noted with other biased GLP-1RAs [11,12], and this divergence has yet to be satisfactorily explained. Differential access to anorectic neurons within the central nervous system due to altered GLP-1R-mediated carriage across the blood brain barrier [40] as well as differential actions of biased ligands on different cell types (“tissue bias”) [41] remain two realistic possibilities to be explored. Nevertheless, it should be highlighted that, in the present report, when administered chronically, [F^1^,G^40^,K^41^.C16 diacid]exendin-4 resulted in superior cumulative reductions in food intake and greater weight loss than [D^3^,G^40^,K^41^.C16 diacid]exendin-4. As [F^1^,G^40^,K^41^.C16 diacid]exendin-4 tended to exert a milder appetite suppressive effect in the acute setting, enhanced tolerability might result while still achieving better weight loss and anti-hyperglycaemic efficacy.

In this study, we did not compare these ligands against class-leading GLP-1RAs such as semaglutide and dulaglutide, and therefore their true therapeutic potential remains undetermined. Nevertheless, our results clearly show that agonists displaying weak acute signalling efficacy and potency can be surprisingly effective for therapeutically important readouts. We also did not test for development of anti-drug antibodies, which are common with exendin-4-derived peptides, albeit seemingly of little therapeutic relevance [42]. A further limitation of our work is that we examined only a limited series of canonical pathways for which a role in GLP-1R signal transduction was already established. Unbiased techniques that simultaneously measure multiple pathways, for example, phosphoproteomic or kinomic analysis, have the potential to enhance our understanding of the unusual pharmacology of biased GLP-1RA ligands and may shed light on new ways to control GLP-1R behaviours.
