# Supplementary material for: Disconnect between signalling potency and in vivo efficacy of pharmacokinetically optimised biased glucagon-like peptide-1 receptor agonists
Source: Mol Metab. 2020 Apr 8;37:100991. doi: 10.1016/j.molmet.2020.100991 (PMC7262448; doi:10.1016/j.molmet.2020.100991)
Supplement: Multimedia component 1 [file mmc1.docx]

**Supplementary Figure 1**

**Supplementary Figure 1. Additional *in vitro* data.** (**A**) Structural features of C16 ligands. (**B**) β-arrestin-2 (βarr2) recruitment in CHO-K1-GLP-1R-β-arrestin-2-EA cells treated with indicated ligand or ligand combination, 30-min incubation, *n*=4, curve fitting of pooled data using method of Stahl (23). (**C**) cAMP responses in INS-1 832/3 cells (10-min incubation with 500 µM IBMX) and MIN6B1 cells (5-min incubation with 500 µM IBMX), both *n*=4 with 4-parameter logistic fit of pooled data shown. (**D**) Quantification of miniG_s_ (mG_s_), miniG_i_ (mG_i_), miniG_q_ (mG_q_) and β-arrestin-2 recruitment by NanoBiT complementation as shown in Figure 1D, calculated after subtracting vehicle response, compared using randomised block one-way ANOVA with Sidak’s test. (**E**) Concentration-response curves for miniG_s_ recruitment to GLP-1R-SmBiT in transiently transfected HEK293T cells (AUC from 30-min stimulation, *n*=5), cAMP in HEK293-SNAP-GLP-1R cells (30-min stimulation, no IBMX, *n*=5), PKA activation via AKAR4-NES FRET assay in HEK293-SNAP-GLP-1R cells (AUC from 30-min stimulation, *n*=6) and β-arrestin-2 recruitment to GLP-1R-SmBiT in transiently transfected HEK293T cells (AUC from 30-min stimulation, *n*=5). Heatmap shows responses normalised to the maximum for each pathway. (**F**) DERET traces indicating GLP-1R internalisation in HEK293-SNAP-GLP-1R cells, *n*=5, pertains to Figure 1E. * p<0.05 by statistical test indicated in the text. Error bars indicate SEM.

**Supplementary Figure 2**

**Supplementary Figure 2. Additional data concerning protein binding of -C16 ligands.** (**A**) Amino acid sequences of exendin-4 and [G^40^,K^41^.C16 diacid]exendin-4 in single letter code. (**B**) cAMP responses in HEK293-SNAP-GLP-1R cells in presence of indicated concentration of FBS, 30-min incubation, *n*=4, 4-parameter logistic fit of pooled data shown. The effect of ± FBS is determined by subtracting logEC_50_ values for each ligand; all comparisons between acylated and non-acylated ligands are non-significant by two-way repeat measures ANOVA with Tukey’s test. (**C**) cAMP and β-arrestin-2 recruitment responses in CHO-K1-GLP-1R-β-arrestin-2-EA cells, 30-min stimulation, *n*=5 with 4-parameter logistic fits of pooled data shown. Error bars indicate SEM.

**Supplementary Figure 3.**

**Supplementary Figure 3. Dose range studies in lean mice.** (**A**) IPGTTs (2 g/kg glucose) performed at the time of or 72 hours after IP administration of the indicated dose of -C16 agonist in lean, male C57Bl/6 mice, *n*=15-16/group, AUCs compared by two-way ANOVA with Sidak’s test. (**B**) Cumulative food intake over 72 hours after IP administration of indicated dose of -C16 agonist in lean, male C57Bl/6 mice, *n*=7-8/group, 1- and 72-hour data plotted separately, two-way repeat measures ANOVA with Tukey’s test. (**C**) Data from (A) and (B) displayed as a dose response analysis with 4-parameter logistic fit shown. * p<0.05 by statistical test indicated in the text. Error bars indicate SEM.

**Supplementary Figure 4**

**Supplementary Figure 4. Repeat sustained administration study.** IPGTT (2 g/kg glucose) performed 72 hours after the final agonist dose in a repeat study with identical protocol to Figure 4, *n*=5 mice/group, time-points compared by two-way repeat measures ANOVA with Tukey’s test (comparison between [F^1^,G^40^,K^41^.C16 diacid]exendin-4 and [D^3^,G^40^,K^41^.C16 diacid]exendin-4 is shown) and AUC compared by one-way ANOVA with Tukey’s test (comparison between [F^1^,G^40^,K^41^.C16 diacid]exendin-4 and [D^3^,G^40^,K^41^.C16 diacid]exendin-4 is shown). * p<0.05 by statistical test indicated in the text. Error bars indicate SEM.

Supplementary Table 1. Unbound fraction in plasma of different species, determined apparent K_D_ values for the binding to plasma proteins and calculated Total Quality Indices.

| Compound | Species | Unbound fraction | App. K_D_ [µM] | TQI |
| --- | --- | --- | --- | --- |
| [K^40^.C16 diacid]exendin-4 | Human | 2.16% / 2.49% | 13.4 / 15.5 | 9.3 / 9.5 |
| [K^40^.C16 diacid]exendin-4 | Mouse | 2.59% / 2.57% | 15.8 / 15.8 | 9.3 / 9.3 |
| [D^3^,K^40^.C16 diacid]exendin-4 | Human | 2.06% / 2.10% | 12.6 / 12.8 | 9.3 / 9.5 |
| [D^3^,K^40^.C16 diacid]exendin-4 | Mouse | 2.32% / 2.43% | 14.2 / 14.8 | 9.3 / 8.9 |
| [F^1^,K^40^.C16 diacid]exendin-4 | Human | 2.16% / 2.18% | 13.4 / 13.4 | 9.3 / 9.5 |
| [F^1^,K^40^.C16 diacid]exendin-4 | Mouse | 2.13% / 2.08% | 12.3 / 12.8 | 8.5 / 9.1 |

The Total Quality Index (TQI) is an artificial value to describe the assay performance. It ranges from 1 to 10 and covers the consistency with the applied binding model, uncertainty of the analysis method and differences in compound recovery at different plasma dilutions. The index can take values between zero and ten. It is calculated as the mean of different quality indices which can also take values between zero and ten (see Supplementary Table 2). A value of 8 or higher is indicating good assay performance.

**Supplementary Table 2. Description of used quality indices**

| Quality index | Description |
| --- | --- |
| Recovery | Significant differences in the recovery rate of the different plasma dilution samples may lead to a distortion of the assay results. Homogenous recovery lead to high QI values. |
| Average QI | Each plasma dilution sample series shall fit to the theoretical model of exclusive single non-covalent binding of the compound to HSA. Poor fit will lead to low QI values. The mean of the quality indices of all plasma dilutions thus describes the applicability of the binding model. |
| 95% confidence interval of f_u_ | Large intra-assay confidence interval shows poor assay performance |
| Number of outlier | High number of statistical outlier show poor assay performance |
| f_b_/f_u_ distance | The mean deviation between measured and calculated f_b_/f_u_ values for each plasma dilution series. Higher deviations indicate poor model fit. |
