# Supplementary material for: Disconnect between signalling potency and in vivo efficacy of pharmacokinetically optimised biased glucagon-like peptide-1 receptor agonists
Source: Mol Metab. 2020 Apr 8;37:100991. doi: 10.1016/j.molmet.2020.100991 (PMC7262448; doi:10.1016/j.molmet.2020.100991)
Supplement: Multimedia component 2 [file mmc2.docx]

# Supplementary Methods

## Cell culture

HEK293-SNAP-GLP-1R cells were maintained in DMEM with 10% foetal bovine serum (FBS), 1% penicillin/streptomycin and G418 (1 mg/ml). HEK293T cells were maintained similarly but without G418. PathHunter CHO-K1-βarr2-EA-GLP-1R cells (DiscoverX) were maintained in F12 medium with 10% FBS, 1% penicillin/streptomycin, G418 (1 mg/ml) and hygromycin (250 µg/ml). INS-1 832/3 cells were maintained in RPMI-1640 with 10% FBS, 1% penicillin/streptomycin, 10 mM HEPES, 1 mM sodium pyruvate and 50 µM β-mercaptoethanol. MIN6B1 cells were maintained in DMEM with 15% FBS and 50 µM β-mercaptoethanol.

## GLP-1R binding affinity measurement

HEK293-SNAP-GLP-1R cells were labelled in suspension with SNAP-Lumi4-Tb (Cisbio, 40 nM) for 1 hour at room temperature in complete medium. After washing and resuspension in Hank’s buffered salt solution (HBSS) containing 0.1% bovine serum albumin (BSA) and metabolic inhibitors to prevent GLP-1R internalisation [20 mmol/L 2-deoxygucose and 10 mmol/L NaN_3_ (18)], cells were treated with 10 nM exendin(9-39)-FITC in competition with a range of concentrations of unlabelled peptide for 24 hours at 4°C before measurement of binding by TR-FRET in a Flexstation 3 plate reader as previously described (17). Binding was quantified as the ratio of fluorescent signal at 520 nm to that at 620 nm, after subtraction of the ratio obtained in the absence of FITC-ligands, and equilibrium binding constants (K_d_) were calculated using Prism 8 (GraphPad Software).

## PKA biosensor assay

HEK293-SNAP-GLP-1R cells were transfected with AKAR4-NES for 36 h prior to the assay and placed in HBSS in 96-well clear-bottom black plates. After a 5-min baseline read at 37 °C (λ_ex_ = 440 nm, λ_em_ = 485 and 535 nm) in a Flexstation 3 plate reader, compounds were injected to each well, and sequential readings immediately commenced. FRET was expressed ratiometrically as signal at 535 nm divided by signal at 485 nm. Individual well responses were normalized to baseline to reduce variability.

## Animal studies

All animal procedures were approved by the British Home Office under the UK Animal (Scientific Procedures) Act 1986 (Project License PB7CFFE7A). Lean male C57Bl/6 mice (8-10 weeks of age, body weight 25-30 g, obtained from Charles River) were maintained at 21-23°C and 12-hour light-dark cycles. *Ad libitum* access to water and normal chow (RM1, Special Diet Services), or diet containing 60% fat to induce obesity and glucose intolerance (D12492, Research Diets) for a minimum of 3 months before experiments, was provided. Mice were housed in groups of four, except for food intake assessments when they were individually caged with one week of acclimatisation prior to experiments.

## Beta cell mass study

Mice were sacrificed and pancreata were dissected and fixed in 4% PFA for 24 h. The tissues were washed twice in PBS and left in 70% ethanol until wax embedding. For each sample, three 5 μm tissue sections separated by 300 μM were stained with guinea pig anti-insulin antibody (ready-to-use, Dako IR002; Alexa Fluor-488 secondary antibody, Invitrogen) and mouse anti-glucagon antibody (1:500, Sigma-Aldrich G2654; Alexa Fluor-568 secondary antibody, Invitrogen). Images were captured using a Zeiss Axio Observer Inverted microscope. Insulin-positive areas were determined using ImageJ as previously described (22) and expressed relative to total pancreas area imaged.

## *ES*calate plasma protein binding assay

Plasma protein binding of [G^40^,K^41^.C16 diacid]exendin-4, [F^1^,G^40^,K^41^.C16 diacid]exendin-4 and [D^3^,G^40^,K^41^.C16 diacid]exendin-4 was determined using the 3BP *EScalate* Equilibrium Shift Assay. In short, the shift of the binding equilibrium of the compound to HSA-coated beads following addition of plasma at various dilutions was analysed. From this concentration-dependent shift, the apparent dissociation constants for binding to HSA on the beads and binding to plasma proteins can be calculated. From the apparent dissociation constant to plasma proteins, the fraction that is not bound to plasma proteins (fraction unbound, f_u_) can be calculated.

**Materials**

Acetonitrile super gradient grade was obtained from VWR, Darmstadt, Germany.

Trifluoroacetic acid LC-MS grade was delivered by Thermo Fisher Scientific, Schwerte, Germany.

Formic acid LC-MS grade and Dimethyl sulfoxide (DMSO) puriss. grade was purchased from Sigma Aldrich, Taufkirchen, Germany.

Water was purified using a Millipore milli-Q A-10 water purification system.

Dulbecco’s phosphate buffered saline (PBS) without Mg^2+^ and Ca^2+^ was obtained from Biochrom GmbH, Berlin, Germany.

**Stock solutions**

1 mM stock solution Ex4-C16: 1.65 mg of the compound was dissolved in 356 µL DMSO.

1 mM stock solution Ex4-asp3-C16: 1.85 mg of the compound was dissolved in 400 µL DMSO.

1 mM stock solution Ex4-phe1-C16: 1.61 mg of the compound was dissolved in 346 µL DMSO.

24 µM stock solutions: 24 µL of the 1 mM stock solution was diluted with 976 µL PBS.

**Plasma stocks**

Human plasma

Pooled normal K2EDTA plasma, BioIVT, Burges Hill, Great Britain

Mouse plasma CD1(ICR)

Pooled CD-1 mouse K2EDTA plasma, BioIVT, Burges Hill, Great Britain

**Plasma dilutions**

Following plasma dilutions were prepared for each assay.

Preparation of plasma dilutions

| Plasma dilution | Plasma volume | PBS volume |
| --- | --- | --- |
| 50% | 200 µl | 200 µl |
| 16.7% | 66.7 µl | 333 µl |
| 5.6% | 22.2 µl | 378 µl |
| 1.9% | 7.41 µl | 393 µl |
| 0.62% | 2.47 µl | 398 µl |

***EScalate* Equilibrium Shift Assay**

Lot: 538-158

The EScalate assay consists of five well lanes with 6 wells containing following concentration of immobilized HSA: 0 µM, 11.4 µM, 20.6 µM, 37.0 µM, 66.7 µM, 120 µM in 85 µl PBS.

**Sample generation**

The Equilibrium Shift Assay samples were generated by addition of 30 µl of the prepared plasma dilutions (50%, 16.7%, 5.6%, 1.9% and 0.62%) and 5 µl of the 24 µM compound stock solution to each well.

A binding protein concentration of 600 µM was assumed for all plasma samples.

After 1 hour of incubation at room temperature under gentle shaking, the kit plate was centrifuged to separate the HSA immobilized on beads from the free plasma proteins. 50 µL of the supernatant were taken as sample from each vial.

For calibration purposes, additional compound solutions in PBS were prepared by diluting the 24 µM stock solution as following:

Preparation of calibration spiking solutions

| Compound concentration | 24 µM stock solution | PBS |
| --- | --- | --- |
| 12 µM | 20 µL | 20 µL |
| 6 µM | 10 µL | 30 µL |
| 3 µM | 5 µL | 35 µL |
| 1.5 µM | 5 µL | 75 µL |

Calibration samples were prepared by spiking 120 µL of the lowest plasma / protein dilution with 5 µL of the compound spiking solution. The calibration samples were incubated together with the assay samples.

**Sample preparation**

Each sample was mixed with 2 µL of the 24 µM stock solution of the chosen internal standard and 250 µL of acidified acetonitrile (containing 1% trifluoroacetic acid) to precipitate the plasma proteins. The samples were centrifuged and 100 µL of the supernatants were diluted with 100 µL of 0.1% aqueous formic acid.

Assignment of internal standards to the test items

| Compound | Chosen internal standard |
| --- | --- |
| [G^40^,K^41^.C16 diacid]exendin-4 | [F^1^,G^40^,K^41^.C16 diacid]exendin-4 |
| [D^3^,G^40^,K^41^.C16 diacid]exendin-4 | [F^1^,G^40^,K^41^.C16 diacid]exendin-4 |
| [F^1^,G^40^,K^41^.C16 diacid]exendin-4 | [D^3^,G^40^,K^41^.C16 diacid]exendin-4 |

**LC-MS analysis**

The samples were analyzed using an Agilent 1290 UHPLC system coupled to an Agilent 6470 triple quadrupole mass spectrometer.

Chromatographic parameters:

Column: Waters Acquity CSH C18, 50 x 2 mm, 1.7 µm

Column temperature: 40°C

Eluent A: 0.1% formic acid in water

Eluent B: acetonitrile

Gradient: 20% B 🡪 80% B in 2.5 min

Flow rate: 800 µL/min

Source: Agilent Jet Stream

Drying gas: 210°C, 13 L/min

Sheath gas: 250°C, 11 L/min

Nebulizer: 35 psig

Capillary voltage: 4.0 kV

Nozzle voltage: 0.5 kV

Acquisition: ESI positive ion mode, MRM

Compound-specific:

| Compound name | Retention time | Fragmentor | Transition / Collision Energy |
| --- | --- | --- | --- |
| [G^40^,K^41^.C16 diacid]exendin-4 | 1.75 min | 190 V | Quantifier: 929.3 🡪 851.8 @ 22V  Qualifier: 929.3 🡪 129.2 @ 43 V |
| [D^3^,G^40^,K^41^.C16 diacid]exendin-4 | 1.78 min | 160 V | Quantifier:926.5 🡪 946.8 @ 31 V  Qualifier: 926.5 🡪 851.8 @ 21 V |
| [F^1^,G^40^,K^41^.C16 diacid]exendin-4 | 1.97 min | 190 V | Quantifier: 1164.0 🡪 851.8 @ 20 V  Qualifier: 1164.0 🡪 754.3 @ 50 V |

**Data analysis**

A quadratic calibration was performed from 0.015 µM to 1 µM using the prepared calibration samples followed by quantitation of the compounds in all samples using the lowest plasma dilution as matrix.

From the determined compound concentrations (APA) of all sample data, the fitting procedure for both dissociation constants was performed according to the following equation.

Function:

$APA=\frac{C_{0}K_{D}^{HSA}\left( K_{D}^{Plasma}+\propto P \right)}{\left[ HSA \right]^{bound}K_{D}^{Plasma}+K_{D}^{HSA}\left( K_{D}^{Plasma}+\propto P \right)}$

where c_0_ is the total concentration of drug in the incubation, K_D_^HSA^ is the dissociation constant of the compound from immobilized HSA on beads, K_D_^Plasma^ is the dissociation constant of the compound from plasma proteins in solution, [HSA]^bound^ is the concentration in solution of HSA bound to the beads, P is the concentration of the binding protein in plasma and α is the applied dilution factor.

Then, the free fraction of the compound in plasma was calculated as follows (assuming a binding protein concentration of 600 µM for all plasma samples).

$f_{u}=\frac{1}{1+\frac{P}{K_{D}^{Plasma}}}$

To evaluate the assay a Total Quality Index (TQI) was calculated to evaluate the consistency with the applied binding model, uncertainty of the analysis method and differences in compound recovery at different plasma dilutions. The index can take values between zero and ten. A value of 8 or higher is indicating good assay performance.
